# Elevated circulating GPHB5 levels in women with insulin resistance and polycystic ovary syndrome: A cross-sectional study and multiple intervention studies

**DOI:** 10.3389/fendo.2022.1010714

**Published:** 2022-12-08

**Authors:** Yanping Wang, Ting Xiang, Xuyun Xia, Hongmin Zhang, Shan Geng, Gangyi Yang, Sheng Qiu, Yirui He, Rui Liu, Ling Li, Hua Liu, Ke Li, Lili Zhang, Zerong Liang, Jianguo He

**Affiliations:** ^1^ Department of Endocrinology, Chongqing Red Cross Hospital (People’s Hospital of Jiangbei District), Chongqing, China; ^2^ Department of Endocrinology, The Second Affiliated Hospital, Chongqing Medical University, Chongqing, China; ^3^ Department of Endocrinology, The First People’s Hospital of Chongqing Liang Jiang New Area, Chongqing, China; ^4^ Department of Endocrinology, The People’s Hospital of Dazu, Chongqing, China; ^5^ The Key Laboratory of Laboratory Medical Diagnostics in the Ministry of Education and Department of Clinical Biochemistry, College of Laboratory Medicine, Chongqing Medical University, Chongqing, China; ^6^ Department of Pediatrics, University of Mississippi Medical Center, Jackson, Mississippi, MS, United States

**Keywords:** GPHB5, bioinformatics, insulin resistance, PCOS, drug intervention

## Abstract

**Objective:**

GPHB5 has been found to be associated with glucose and lipid metabolism in animal studies. However, the association of GPHB5 with IR and metabolic disorders remains unknown, and there is a lack of research in humans. Our aim in this study was to investigate the relationship between circulating GPHB5 and metabolic disorders in humans.

**Methods:**

Bioinformatics analysis was performed to understand the relationship between GPHB5 and metabolic disorders. GPHB5 mRNA expression in mice and rats was determined using RT-qPCR. Circulating GPHB5 concentrations were measured with an ELISA kit. EHC and OGTT were performed in humans.

**Results:**

Bioinformatics analysis shows that GPHB5 is associated with metabolic disorders and PCOS. GPHB5 mRNA expression levels in the metabolic-related tissues of HFD-fed mice, db/db and ob/ob mice, and PCOS rats were significantly higher than those of WT mice or rats. In human studies, we find that circulating GPHB5 levels were significantly higher in women with IR and PCOS. GPHB5 levels were positively correlated with age, BMI, WHR, BP, FBG, 2 h-BG, FIns, 2 h-Ins, TC, LDL-C, HbA1c, and FFA, but negatively correlated with adiponectin. Furthermore, GPHB5 was positively correlated with DHEAS and FAI, while negatively correlated with SHBG, FSH, SHBG and FSH. The increased GPHB5 concentration was related to IR and PCOS. After the treatment of metformin, GLP-1RA (Lira), and TZDs, circulating GPHB5 levels were decreased.

**Conclusions:**

Our results reveal that circulating GPHB5 could be a biomarker and potential therapeutic target for IR and PCOS in women.

## Introduction

The incidence of polycystic ovary syndrome (PCOS) is approximately 10% in women of child-bearing age and is characterized by oligovulation or anovulation, presence of polycystic ovary, hyperandrogenism, etc. Although the current diagnostic criteria for PCOS do not include insulin resistance (IR) and metabolic disorders, they have a prominent role in the pathogenesis of PCOS. Approximately 70% of women with PCOS have IR and metabolic disorders (1–3). PCOS is not only associated with reproductive problems but also leads to obesity and metabolic diseases, such as type 2 diabetes mellitus (T2DM), metabolic syndrome (MetS), atherosclerosis, and cardiovascular diseases. etc (4–7). Although extensive research has been conducted on PCOS, its pathogenesis is still not understood (8). In recent years, in patients with PCOS, some cytokines have been found to be dysregulated and related to IR, including leptin, adiponectin, RBP4, and irisin (2, 9). Therefore, an in-depth study of these cytokines is helpful to provide new approaches for the diagnosis and treatment of PCOS.

The glycoprotein hormone family consists of a variety of hormones, including follicle- stimulating hormone (FSH), luteinizing hormone (LH), thyroid hormone (TSH), etc. They have a variety of physiological functions, including reproduction and energy metabolism (10, 11). These glycoprotein hormones are composed of two glycoprotein subunits containing a cystine structure, α subunit (GPHA1) and β subunit (GPHB1-4). The common structure of these hormones is the α-subunit, and for each hormone, the ß-subunit is a unique structure (12).

In 2002, two new glycoprotein hormone subunits, glycoprotein α2 (GPHA2) and glycoprotein β5 (GPHB5), were identified in the human genome (10). They are activated by binding to thyrotropin (TSH) receptors (TSHRs) (11). Therefore, GPHA2 and GPHB5 were named as thyrostimulin to distinguishing from TSH (12, 13). It has been found that GPHB5 and GPHA2 can form a heterodimer (GPHB5/GPHA2) and are secreted into the blood to participate in reproduction, tumor cell proliferation, skeletal development, and immune and inflammatory regulation (14–16). A previous study in mice found that GPHB5 overexpression improved diet-induced obesity, reduced the levels of blood glucose, triglycerides, cholesterol and insulin, and increased the levels of T3 and T4 (17), suggesting that it is involved in the process of energy equilibrium *in vivo*. But, the relationship between GPHB5 and metabolic diseases as well as its role has not been reported in humans.

In this study, we measured, for the first time, the circulating GPHB5 concentration in PCOS and IR women and compared it with controls. In addition, we investigated whether the circulating GPHB5 concentration was regulated by blood glucose, insulin and free fatty acids (FFAs). Ultimum, we evaluated the effect of metformin, GLP-1RA (Lira), and TZD treatment on circulating GPHB5 levels in patients with PCOS.

## Research design and methods

### Bioinformatics analysis

Gene Expression Profiling Interactive Analysis (GEPIA2) was used for the analysis of GPHB5 differentially expressed genes (DEGs), survival analysis and related genes in cancers. The top 100 DEGs of TSHR were mapped into the Search Tool or the Retrieval of Interacting Genes (STRING) database (v11.0) to build a protein-protein interaction (PPI) network. On the basis of THSR analysis, GPHB5, an important ligand for TSHR, was further analyzed for IR. We obtained the GSE34526 dataset, which is the expression profile of PCOS and normal individuals for the granulosa cell array. We analyzed data from 7 PCOS individuals and divided them into two groups according to the average value of GPHB5 expression. Limma software package was used for DEGs analysis. The DEGs between 3 high expression samples and 4 low expression samples were filtered. A log2 fold change > 1.5 and p value< 0.05 were considered obvious DEGs. The cluster Profiler package was used for Gene Ontology (GO) and Kyoto Encyclopedia of Genes and Genomes (KEGG) pathway analyses. Visualization of the PPI network was constructed by Cytoscape (18).

### Population cohort study

**Figure 1:**
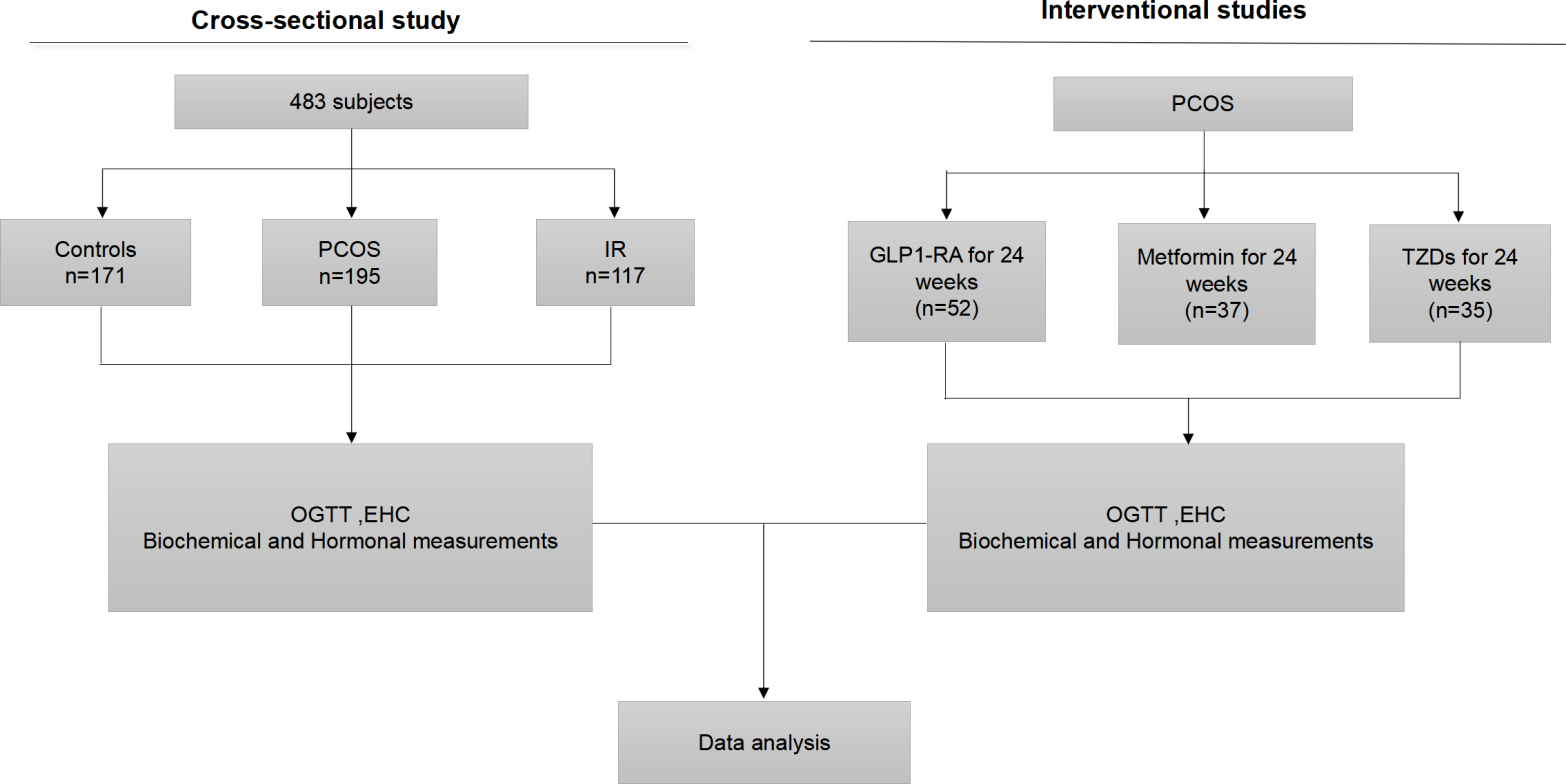
Experimental design. OGTT, oral glucose tolerance test; EHC, euglycemic- hyperinsulinemic clamp; GLP1-RA, glucagon-like peptide-1 receptor agonists; TZD, rosiglitazone.

A total of 483 individuals participated in this cross-sectional cohort and/or intervention study, including 195 women with PCOS, 117 women with IR and 171 normal controls, with an average age of 27 (24 - 31) years. All women with PCOS and IR were from the Department of Endocrinology or Reproduction of the Second Affiliated Hospital of Chongqing Medical University or Department of Endocrinology, Chongqing Red Cross Hospital (**Figure 1**). The diagnosis of PCOS was determined according to the 2003 Rotterdam consensus standard (19). The presence of at least two of the following three features: 1) oligo-amenorrhea or chronic anovulation; 2) clinical and/or biochemical hyperandrogenism; 3) ultrasound appearance of polycystic ovaries. Exclusion criteria included other known hyperandrogenaemia and ovulation dysfunction, such as congenital adrenal hyperplasia, Cushing’s syndrome, androgen secreting tumors, thyroid diseases and hyperprolactinemia. During the EHC experiment, when the M-value was< 5.6 mg/kg/min, women were considered to have IR, as previously reported (20). All women with PCOS and IR were newly diagnosed, did not use any treatment or had stopped taking drugs for more than three months. A total of 171 age-matched normal controls were recruited from routine physical examination or from community or school. Individuals with T2DM or T1DM, or other diseases or using any drugs were excluded from this study. Women were given written informed consent to participate in this study. This study was performed according to the Declaration of Helsinki and approved by the ethical committee of Chongqing Medical University. This study was registered at the Chinese Clinical Trial Registry (CHICTR- OCS-13003185).

### Anthropometric and biochemical measurements

Body mass index (BMI) was determined by weight and height. The homeostasis model evaluation of insulin resistance (HOMA-IR) was determined by the following formula: fasting insulin (FIns, mU/mL) × fasting blood glucose (FBG, mmol/L)/22.5 (20). Body fat (Fat%) was measured by bioelectrical impedance (BIA-101; RJL Systems). The glucose-oxidase method and anion-exchange HPLC were used to determine blood glucose or glycosylated hemoglobin (HbA1c). Insulin was determined by chemiluminescence. FFAs and blood lipids were measured with a commercial kit (Randox Laboratories, Antrim, U.K.) and an autoanalyzer, respectively (21).

### OGTTs and EHCs

Oral glucose tolerance tests (OGTTs) were performed at 8 AM in the morning, 12 hours after fasting, subjects were given 75 g glucose and blood samples were collected at 0, 30, 60 and 120 min to determine glucose, insulin and GPHB5 levels.

Euglycemic-hyperinsulinemic clamp (EHC) studies were performed in all individuals as previously published (18). Briefly, the individuals were fasted for 12 h. At 8:00 AM, the catheters were inserted into the left and right anterior elbow veins to collect blood, and infused 20% glucose and insulin. During the EHC, human insulin (1 mU/kg/min) was infused for 2 h, and 20% glucose was infused at a variable rate to maintain blood glucose at fasting levels. The EHC experiment lasted for 120 min, and blood glucose was detected every 15 min. The glucose infusion rate (GIR) was defined as the M value (mg/kg per min) during the stable period of clamping. Blood samples were obtained at 0, 80, 100 and 120 min for GPHB5 and insulin measurements. Serum was stored at - 80°C until use.

### Drug intervention study

A total of 124 women with PCOS were randomly divided into three groups and received metformin (n = 37) or glucagon-like peptide-1 receptor agonists (GLP-1-RA) (n = 52) or rosiglitazone (n = 35) treatment for 6 months. Individuals were excluded from participation if they were pregnant or planning to become pregnant. All women were given informed written consent to the side effects of these drugs at the beginning of the study. In the first subgroup, 37 patients with PCOS received metformin treatment for 24 weeks. The dose of metformin was increased from 0.5g to 1g twice daily. In the second subgroup, 52 patients received liraglutide (liar) treatment for 12 weeks. The dose of liar was increased from 0.6 mg to 1.8 mg once daily. In the third subgroup, 35 patients received rosiglitazone (TZD) treatment for 12 weeks. The dose of TZD was increased from 0.4 mg to 0.8 mg once daily. At the end of 3rd month and the end of treatment, biochemical parameters and GPHB5 concentration were measured and OGTT and EHC were performed. Blood samples were obtained per-treatment, at week 12 and at the end of treatment, as well as at the indicated time points during the OGTT and EHC.

### Sex hormone measurement

Serum hormonal measurements including luteinizing hormone (LH), follicle-stimulating hormone (FSH), testosterone, testosterone (TEST), sex hormone binding globulin (SHBG), and dehydroepiandrostenedione sulfate (DHEAS) were determined as previously reported (22).

### Cytokine concentration determination

We used an ELISA kit to measure circulating GPHB5 levels according to the manufacturer’s instructions (Kete Biotechnology Co., Ltd, Jiangshu, China). This kit has high sensitivity and specificity for the measurement of GPHB5 without obvious cross-reactivity. The intra- and interassay coefficients of variation (CVs) were< 10% and< 8%, respectively. As previously published (23), serum adiponectin (Adipoq) concentrations were measured by an ELISA (sk00010-01, Aviscerabioscience Inc., USA), with intra- and inter-assay CVs of 4% - 8% and 8% - 12%, respectively.

### Animal study

Eight-week-old male C57BL/6J (WT), db/db, and ob/ob mice were fed a normal chow diet (NCD, 11% fat) for 4 weeks. To establish nutrition-related obesity, mice were fed a NCD or high-fat diet (HFD) (45% fat, #MD 12032, Medicine Inc. Jiangsu, China) for 12 weeks. To establish the PCOS animal model, rats were subcutaneously injected with dehydroepiandrosterone (DHEA, 60mg/kg/day, # H10940064, Yangzhou Pharmaceutical Co., Ltd) daily for 35 consecutive days and fed a NCD or HFD for 12 weeks. Mice or rats were euthanized by isoflurane. Tissues were collected and frozen immediately in liquid nitrogen for mRNA expression analysis.

### mRNA expression analysis

Real-time quantitative PCR (RT-PCR) was performed as previously described using β-actin as a control gene (23). The primer pairs used were F: 5’-CCAGACAG GTGACAGTGAAGC-3’ and R: 5’- ACATCGGACAGCCATAGGG-3’ for GPHB5, and 5’-CCCTGAACCCTAAGG CCAACCGTGAA AA-3’ and 5’-TCTCCGGAGTCCATCACAATGCCTGTG-3’ for ß-actin.

### Statistical analysis and calculations

The free androgen index (FAI) was calculated as (testosterone/SHBG)×100 (24). The glucose (AUC_g_) and insulin (AUC_i_) areas under the curve were calculated using the trapezoidal rule. Body adiposity index (BAI) was calculated as [hip circumference/height^1.5^ – 18]. Visceral adiposity index (VAI) = WC/[36.58 + (1.89 ×BMI] × (TG/0.81 × 1.52/HDL- C] (25). Data are expressed as the mean ± SD or median with inter quartile range. All statistical analyses were performed by using the SPSS software package (version 2.0, SPSS Inc.). A two-tailed *p* value< 0.05 was considered statistically significant. A Kolmogorov-Smirnov test was used to analyze whether each variable was normally distributed. Nonnormally distributed data were logarithmically transformed before analysis. The correlation between GPHB5 and other anthropometric and biochemical indexes was analyzed based on linear regression analysis. Binary logistic regression models were employed to control for possible confounding variables and to evaluate the relationship between GPHB5 and the occurrence of PCOS and IR. To determine the impact size and the value of using circulating GPHB5 to predict PCOS, receiver operating characteristic (ROC) curve analyses were performed. The differences between multiple groups were analyzed by one-way ANOVA, followed by a *post hoc* PLSD test for the comparison between two groups. The equation: $n={\left(\frac{Z_{\alpha /2}\sigma }{\epsilon \mu }\right)}^{2}$ (σ, standard; µ, mean; Z_α/2_ = 1.96, α = 0.05, ϵ = 10%) was used to estimate the sample size (26).

## Results

### Bioinformatics analysis

**Figure 2:**
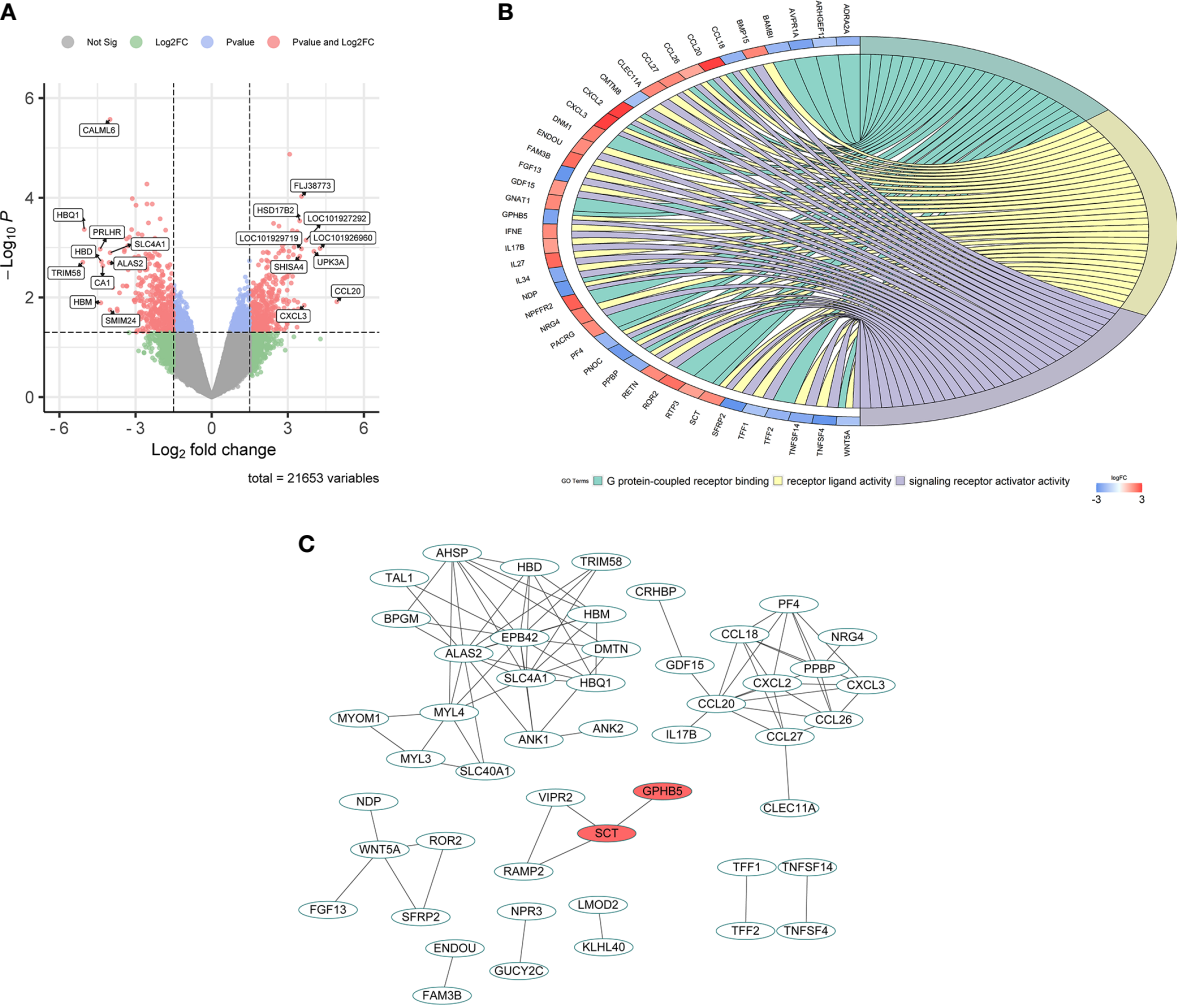
Bioinformatic analysis for GPHB5 related genes and signaling pathways in PCOS individuals. **(A)** Volcano map of DEGs for GPHB5 high expression and low expression. **(B)** GO enrichment analysis of DEGs. **(C)** PPI network of DEGs.

In the GSE34526 dataset, 7 PCOS patients were divided into an high GPHB5 expression group and a low GPHB5 expression group according to the gene expression profile of blood granulosa cells. 853 DEGs were obtained from the analysis of DEGs between the two groups (**Figure 2A**). GO enrichment analysis found that GPHB5 was involved in G protein-coupled receptor binding, receptor and ligand signaling activation and other molecular functions. Genes related to glucose and lipid metabolism, such as adrenoceptor alpha 2A (ADRA2A), growth differentiation factor 15 (GDF15), BMP and activin membrane bound inhibitor (BAMBI), were enriched in the above signaling pathways (**Figure 2B**). PPI network analysis showed that secretin (SCT) interacted with GPHB5 (**Figure 2C**). It has been widely reported that STC plays a role in adipose tissue browning, appetite change and insulin sensitivity (27, 28). Therefore, these data preliminarily suggest that GPHB5 is related to metabolic disorder and PCOS.

### GPHB5 mRNA expression in mice

**Figure 3:**
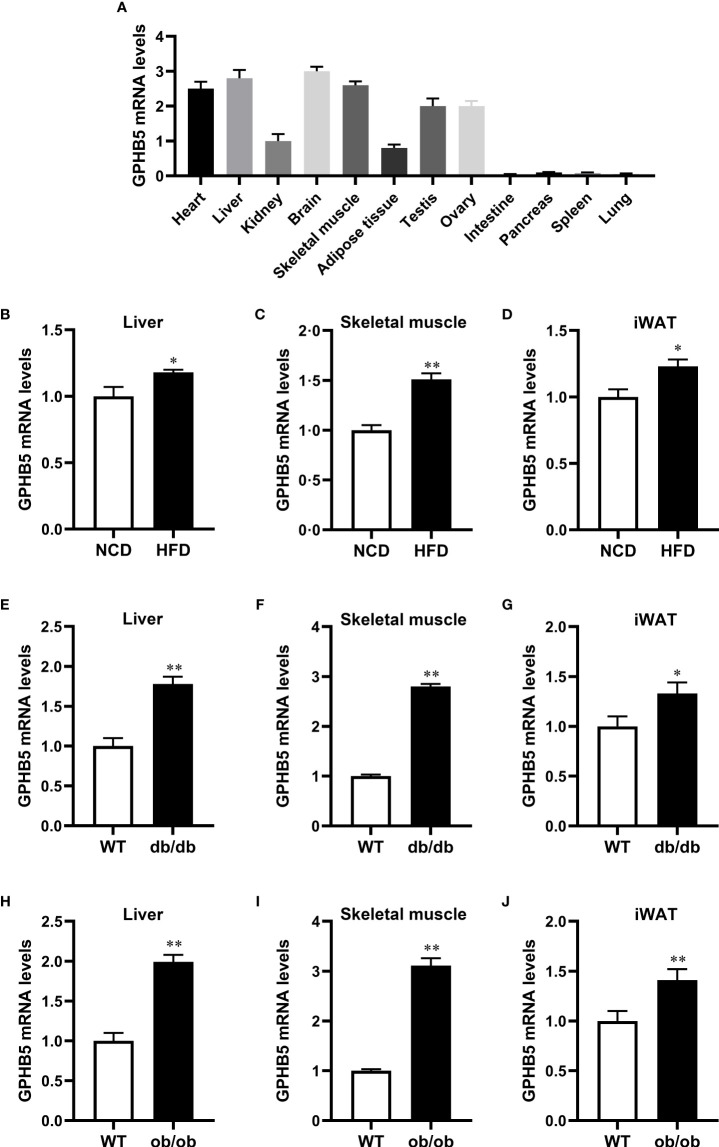
GPHB5 mRNA expression in mice. **(A)** GPHB5 mRNA expression in various tissues of mice. **(B)** GPHB5 mRNA expression in the liver of NCD- or HFD-fed mice. **(C)** GPHB5 mRNA expression in the skeletal muscle of NCD- or HFD-fed mice. **(D)** GPHB5 mRNA expression in the inguinal white adipose tissue (iWAT) of NCD- or HFD-fed mice. **(E)** GPHB5 mRNA expression in the liver of WT or db/db mice. **(F)** GPHB5 mRNA expression in the skeletal muscle of WT or db/db mice. **(G)** GPHB5 mRNA expression in the iWAT of db/db or mice. **(H)** GPHB5 mRNA expression in the liver of WT or ob/ob mice. **(I)** GPHB5 mRNA expression in the skeletal muscle of WT or ob/ob mice. **(J)** GPHB5 mRNA expression in the iWAT of WT or ob/ob mice. Data are means ± SD. *p < 0.05; **p < 0.01 *vs.* NCD or WT.

To further analyze whether GPHB5 is related to metabolism and IR, we performed tissue expression analysis in normal and obese mice. RT-PCR analysis showed that GPHB5 was highly expressed in heart, liver, brain, skeletal muscle, testis and ovary tissues (**Figure 3A**). Importantly, in HFD-fed mice, NCD-fed db/db and ob/ob mice, GPHB5 mRNA expression in metabolism-related tissues, including skeletal muscle, fat and liver, was significantly higher than that in WT mice (**Figures 3B–J**). These results suggest that GPHB5 is related to metabolism.

**Figure 4:**
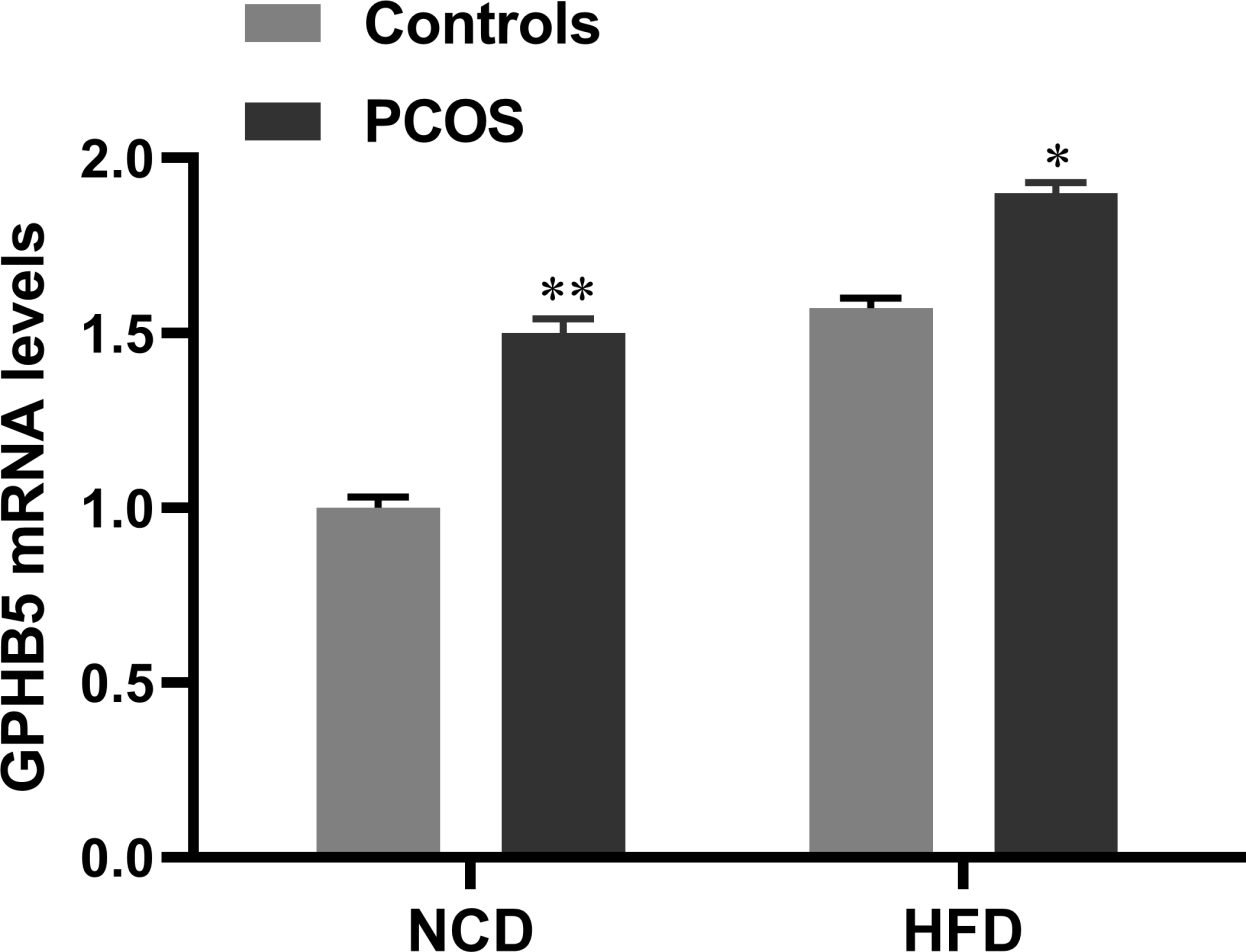
mRNA expression of GPHB5 in the liver of NCD- or HFD-fed WT or PCOS rats. Data are means ± SD. *p<0.05; **p< 0.01 *vs.* controls.

To investigate the effect of androgen on GPHB5, we established a PCOS rat model by subcutaneous injection of DHEA. RT-PCR analysis found that GPHB5 mRNA expression was significantly increased in the livers of both NCD- and HFD-fed PCOS rats (**Figure 4**). Thus, these results indicate that metabolic disorders and androgens may stimulate the secretion and release of GPHB5 *in vivo*.

### Circulating GPHB5 concentration in newly diagnosed PCOS and IR women

All basic parameters of the three groups of study individuals are shown in **Table 1**. In newly diagnosed PCOS and IR women, blood pressure, glucose metabolism-related indicators, including FBG, 2 hour blood glucose after glucose challenge (2-h OGTT) and HbA1c levels, obesity-related indicators, including BMI and WHR, and blood lipids, including total cholesterol (TC), triglycerides (TG) and low-density lipoprotein cholesterol (LDL-C), were significantly higher than those in normal women, while HDL-C was significantly lower (**Table 1**). Furthermore, in PCOS and IR women, insulin levels (including fasting insulin and 2-hour OGTT insulin) and HOMA-IR were also significantly higher than those in the control population. Compared with PCOS individuals, IR individuals had higher diastolic blood pressure (DBP), FBG, FFA and LDL-C (**Table 1**).

**Table 1:** Main clinical features and circulating GPHB5 levels in all study population.

Variable	Controls (n = 171)	PCOS (n = 195)	IR (n = 117)
Age (years)	26 (24 - 28)	26 (23 - 30)	27 (24 - 33)
GPHB5 (μg/L)	2.35 (1.77 - 3.16)	3.64 (3.09 - 4.52)**	4.13 (3.31 - 4.74)**
Adiponectin (μg/L)	46.5 ± 14.8	26.2 ± 12.8**	21.7 ± 13.4**
BMI (kg/m2)	20.2 (18.7 - 22.1)	25.8 (22.8 - 28.7)**	25.5 (24.0 - 28.4)**
WHR	0.78 (0.74 - 0.82)	0.87 (0.82 - 0.92)**	0.87 (0.82 - 0.91)**
SBP (mmHg)	106 (100 - 112)	118 (108 - 121)**	116 (109 - 128)**
DBP (mmHg)	71 (64 - 78)	71 (67 - 79)	75 (69 - 84)**^▴^
FBG (mmol/L)	4.52 (4.31 - 4.89)	5.32 (4.97 - 5.71)**	5.43 (5.11 - 5.95)**
2 h-BG (mmol/L)	5.05 (4.46 - 5.79)	7.91 (6.63 - 9.03)**	8.09 (6.82 - 9.29)**
FIns (mU/L)	7.06 (5.94 - 9.19)	18.49 (13.12 - 29.79)**	18.33 (12.02 - 25.75)**
2 h-Ins (mU/L)	27.1 (17.6 - 52.5)	145.4 (103.1 - 235.6)**	140.6 (98.1 - 282.9)**
TG (mmol/L)	0.98 (0.70 - 1.68)	1.53 (1.11 - 2.21)**	1.44 (0.64 - 1.59) **
TC (mmol/L)	4.02 (3.42 - 4.52)	4.64 (4.01 - 5.12)**	5.10 (4.52 - 5.50)**^▴▴^
HDL-C (mmol/L)	1.29 (1.07 - 1.52)	1.17 (1.00 - 1.33)**	1.18 (0.96 - 1.54)**
LDL-C (mmol/L)	2.29 (1.80 - 2.69)	2.61 (2.10 - 3.19)**	3.20 (2.54 - 4.55)**^▴▴^
FFA (μmol/L)	0.50 (0.36 - 0.70)	0.52 (0.40 - 0.68)	0.81 (0.52 - 1.11)**^▴▴^
HbA1c	5.10 (5.00 - 5.30)	5.50 (5.20 - 5.70)**	5.42 (5.10 - 5.67)**
AUCg	9.08 ± 1.57	12.90 ± 2.47**	13.20 ± 2.76**
AUCi	78.1 (48.3 - 106.9)	196.3 (142.3 - 294.9)**	181.0 (124.3 - 275.7)**
HOMA-IR	1.40 (1.16 - 1.94)	4.49 (3.03 - 7.02)**	4.50 (2.93 - 6.50)**
VAI	1.43 (0.93 - 2.37)	2.42 (1.69 - 3.71)**	1.64 (0.86 - 2.83)^▴▴^

BMI, body mass index; WHR, waist-to-hip ratio; SBP, systolic blood pressure; DBP, diastolic blood pressure; TC, total cholesterol; TG, triglyceride; HDL-C, high-density lipoprotein cholesterol; LDL-C, low-density lipoprotein cholesterol; FFA, free fatty acids; FBG, fasting blood glucose; 2 h-BG, 2h blood glucose after glucose overload; Fins, fasting insulin; 2h-Ins, 2h insulin after glucose overload; HOMA-IR: HOMA-insulin resistance index; AUCi, the area under the curve for insulin; AUCg, the area under the curve for glucose; VAI, visceral adiposity index; Values were given as means ± SEM or median (interquartile Range). **p < 0.01 compared with Controls; ^▴^p < 0.05, ^▴▴^p < 0.01 compared with PCOS.

**Figure 5:**
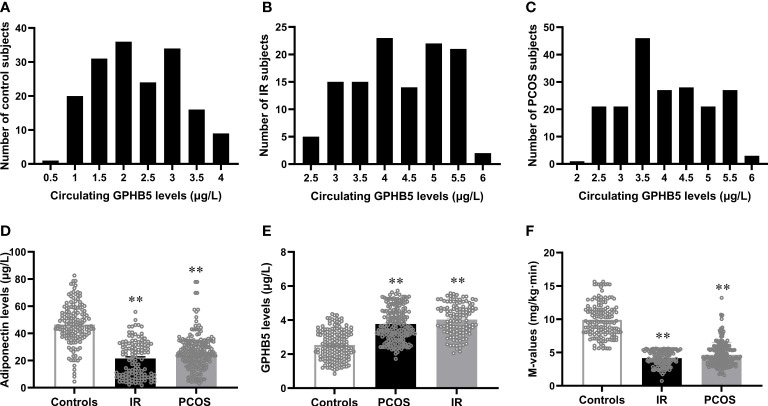
Circulating GPHB5 and adiponectin levels and M-values in the study population. **(A-C)** Distribution range of circulating GPHB5 concentration in the controls **(A)**, IR **(B)** and PCOS **(C)** individuals. **(D)** Circulating adiponectin levels in three groups. **(E)** Circulating GPHB5 levels in three groups. **(F)** M-values during the EHC. Data are means ± SD. **p < 0.01 *vs.* controls.

To explore the relationship between GPHB5 and IR and PCOS, we next measured the levels of serum Adipoq and GPHB5 in the study population. We first analyzed the distribution range of circulating GPHB5 concentrations in all study populations and found that the GPHB5 concentration range of normal controls was mainly 1.0 - 3.5 μg/L, that of the IR population was 3.0 - 5.5 μg/L, and that of the PCOS individuals was 2.5 - 5.5 μg/L (**Figures 5A–C**). Importantly, serum Adipoq levels were significantly lower, whereas GPHB5 levels were higher in women with IR and PCOS than in normal women (**Table 1**; **Figures 5D, E**). Accordingly, M-values in IR and PCOS women during the EHC were significantly lower than those in normal controls (5.54 ± 0.65 for IR, 4.65 ± 1.82 for PCOS *vs.* 9.90 ± 2.56 mg/kg/min) (**Figure 5F**). These data further suggest that GPHB5 is associated with hypoadiponectin and decreased insulin sensitivity in IR and PCOS populations.

### Comparison of sex hormone levels in the study population

In PCOS and IR individuals, DHEAS and FAI were significantly higher than those in the control group, while SHBG and FSH were significantly lower. Compared with the control group, the levels of TEST and LH in the PCOS group were significantly higher and PRL was lower, while these hormones decreased or remained unchanged in the IR group (**Table 2**). These results indicate that PCOS women have hormone disorders, especially hyperandrogenemia.

**Table 2:** Main hormone levels in all study populations.

Variable	Controls (n = 156)	PCOS (n = 195)	IR (n = 116)
SHBG (nmol/L)	56.6 (41.7 - 72.2)	32.6 (23.5 - 46.0)**	35.0 (25.1 - 51.9)**	
DHEAS (μg/dl)	184.0 (146.1 - 222.3)	237.3 (173.1 - 315.9)**	227.9 (172.7 - 302.9)**	
E2 (pg/ml))	46.9 (26.0 - 67.5)	41.7 (31.7 - 56.7)	45.3 (33.1 - 55.2)	
TEST (nmol/L)	1.66 (1.19 - 2.11)	2.08 (1.39 - 2.56)**	1.31 (1.06 - 1.86)*^▴▴^	
LH (IU/L)	4.55 (3.23 - 6.44)	7.58 (4.14 - 11.57)**	4.00 (2.97 - 6.14)^▴▴^	
FSH (IU/L)	7.56 (6.52 - 9.27)	6.18 (5.33 - 7.40)**	6.46 (5.29 - 7.95)**	
PRL (μIU/mL)	318.0 (225.9 - 404.7)	265.4 (188.5 - 367.4)*	288.0 (217.3 - 417.6)	
FAI	2.76 (1.79 - 4.47)	6.22 (3.72 - 9.04)**	3.81 (2.36 - 6.23)**^▴▴^	

FAI, free androgen index; LH, luteinizing hormone; FSH, follicle-stimulating hormone; DHEA-S, dehydroepiandrosterone-sulfate; SHBG, sex hormone-binding globulin; TEST, testosterone; E2, estrogen. Values were given as means ± SEM or median (interquartile Range). *p < 0.05, **p < 0.01 compared with Controls; ^▴▴^p < 0.01 compared with PCOS.

### The relationship between GPHB5 and metabolic parameters, hormones, IR and PCOS in all study populations

**Figure 6:**
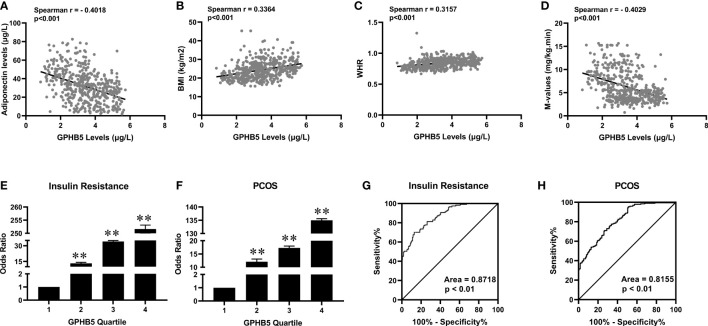
The relationship between GPHB5 and obesity, IR and PCOS in the study population. **(A)** Correlation between circulating GPHB5 and adiponectin. **(B)** Correlation between circulating GPHB5 and BMI. **(C)** Correlation between circulating GPHB5 and WHR. **(D)** Correlation between circulating GPHB5 and M-values. **(E, F)** The odds ratio of having IR **(E)** and PCOS **(F)** in different quartiles of circulating GPHB5. **(G, H)** ROC curve analyses for the prediction of IR **(G)** and PCOS **(H)** according to circulating GPHB5 levels. Data are means ± SD. **p < 0.01. vs. quartile 1.

**Table 3** shows the correlation analysis of GPHB5 with other variables and Adipoq. We found that circulating GPHB5 levels were significantly positively correlated with age, BMI, WHR, BP, FBG, 2 h-BG, FIns, 2 h-Ins, TC, LDL-C, HbA1c, FFA and HOMA-IR, but negatively correlated with Adipoq and M-values (**Figures 6A–D**; **Table 3**). In addition, GPHB5 was positively correlated with DHEAS and FAI, while negatively correlated with SHBG and FSH (**Table 3**). Multivariate linear regression analyses showed that FBG, FIns, FFA and Adipoq were independent influential factors for circulating GPHB5 (**Table 3**). The regression equation is as follows: Y_GPHB5_ = -17.89 + 0.31 × FBG + 1.05 × FIns + 0.14 × FFA - 0.18 × Adipoq (R^2^ = 0.305). Next, we further analyzed the change trend of GPHB5 concentration by using the row mean score difference and the Cochran-Armitage trend test, and found that the increased GPHB5 concentration was related to IR and PCOS (**Table 4**).

**Table 3:** Correlation analysis of variables associated with circulating GPHB5 levels in the population.

	Simple		Multiple	
Variable	*r*	*P*	*r*	*P*
Age (years)	0.131	< 0.01		
BMI (kg/m^2^)	0.336	< 0.01		
WHR	0.316	< 0.01		
SBP (mmHg)	0.174	< 0.01		
DBP (mmHg)	0.297	< 0.01		
FBG 0min(mmol/L)	0.289	< 0.01	0.31	< 0.01
2 h-BG (mmol/L)	0.368	< 0.01		
FIns (mU/L)	0.255	< 0.01	1.05	< 0.01
2 h-Ins (mU/L)	0.298	< 0.01		
SHBG (nmol/L)	-0.170	< 0.01		
DHEAS (μg/dl)	0.142	< 0.01		
E2 (pg/ml)	0.025	0.592		
TEST (nmol/L)	0.065	0.16		
LH (IU/L)	0.076	0.101		
FSH (IU/L)	-0.134	< 0.01		
PRL (μIU/mL)	-0.047	0.311		
FAI	0.181	< 0.01		
TC (mmol/L)	0.242	< 0.01		
TG (mmol/L)	0.073	0.113		
HDL-C (mmol/L)	0.001	0.975		
LDL-C (mmol/L)	0.233	< 0.01		
FFA (μmol/L)	0.202	< 0.01	0.14	< 0.01
HbA1c	0.238	< 0.01		
M-value (mg/kg/min)	0.397	< 0.01		
HOMA-IR	0.242	< 0.01		
Adiponectin (μg/L)	-0.402	< 0.01	-0.18	< 0.01

**Table 4:** Row Mean Scores and Cochran-Armitage Trend test of the impact of circulating GPHB5 levels on IR and PCOS.

	Insulin resistance		PCOS	
Model adjusted	χ^2^	*p-*value	χ^2^	*p-*value
Row Mean Scores test	1181.788	< 0.0001	1444.192	< 0.0001
Cochran-Armitage Tread Test	118.263	< 0.0001	128.429	< 0.0001

To further reveal the association between GPHB5 and IR and PCOS, we divided the circulating GPHB5 concentration into four titers (tertile 1,< 2.17 μg/L; tertile 2, 2.17 to 3.16 μg/L; tertile 3, 3.16 to 3.95 μg/L; tertile 4, > 3.95 μg/L for IR and tertile 1,< 2.35 μg/L; tertile 2, 2.35 to 3.17 μg/L; tertile 3, 3.17 to 3.96 μg/L; tertile 4, > 3.96 μg/L for PCOS). When the circulating GPHB5 concentration was in tertiles 2, 3 and 4, the odds ratios (ORs) of having IR and PCOS were 13.2 (95% confidence interval (CI), 2.60; 2.74), 34 (95% CI, 3.44; 3.55) and 251.6 (95% CI, 4.59; 4.82) for IR (**Figure 6E**), and 12.10 (95% CI, 2.71; 2.83), 17.3 (95% CI, 3.44; 3.64) and 135 (95% CI, 4.59; 4.80) for PCOS (**Figure 6F**).

To explore the predictive effect of GPHB5 on IR and PCOS, we performed ROC curve analysis. The results showed that the best cut-off value for circulating GPHB5 to predict IR was 3.51 µg/L (sensitivity 70.1%, specificity 87.1%, and AUC 0.87; **Figure 6G**), and to predict PCOS was 2.37 μg/L (sensitivity 95.4%, specificity 58.8%, and AUC 0.82, **Figure 6H**).

### Circulating GPHB5 concentration in interventional studies

**Figure 7:**
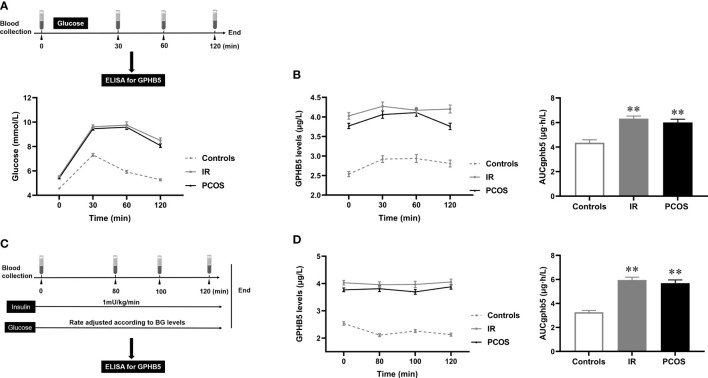
Circulating GPHB5 concentration in interventional studies. **(A)** Experimental procedure for the OGTT and blood glucose during the OGTT. **(B)** GPHB5 concentration and AUC_GPHB5_ during the OGTT. **(C)** Experimental design for the EHC. **(D)** GPHB5 concentration and AUC_GPHB5_ during the EHC. Data are means ± SD. **p < 0.01 *vs.* controls.

To further investigate the relationship between circulating GPHB5 and blood glucose and serum insulin, we conducted OGTT and EHC studies and observed changes in circulating GPHB5 levels in the study population (**Figures 7A, C**). There was no significant change in circulating GPHB5 levels stimulated by elevated blood glucose and insulin levels during the OGTT. However, AUC_GPHB5_ in women with IR and PCOS was significantly higher than that in controls (**Figure 7B**). Furthermore, there was no significant change in circulating GPHB5 levels stimulated by elevated insulin during the EHC. However, during the clamping, AUC_GPHB5_ in IR and PCOS women was significantly higher than that of the controls (**Figure 7D**). During the OGTT, there was no significant difference in GPHB5 levels at each time point in IR, PCOS and control individuals compared with the baseline (**Figure 7B**). During EHC, high insulin levels did not lead to changes in GPHB5 levels (**Figure 7D**). These results indicate that the circulating GPHB5 concentration is not affected by blood glucose and insulin levels.

### GLP-1RA, metformin and rosiglitazone treatment increases circulating GPHB5 levels

**Figure 8:**
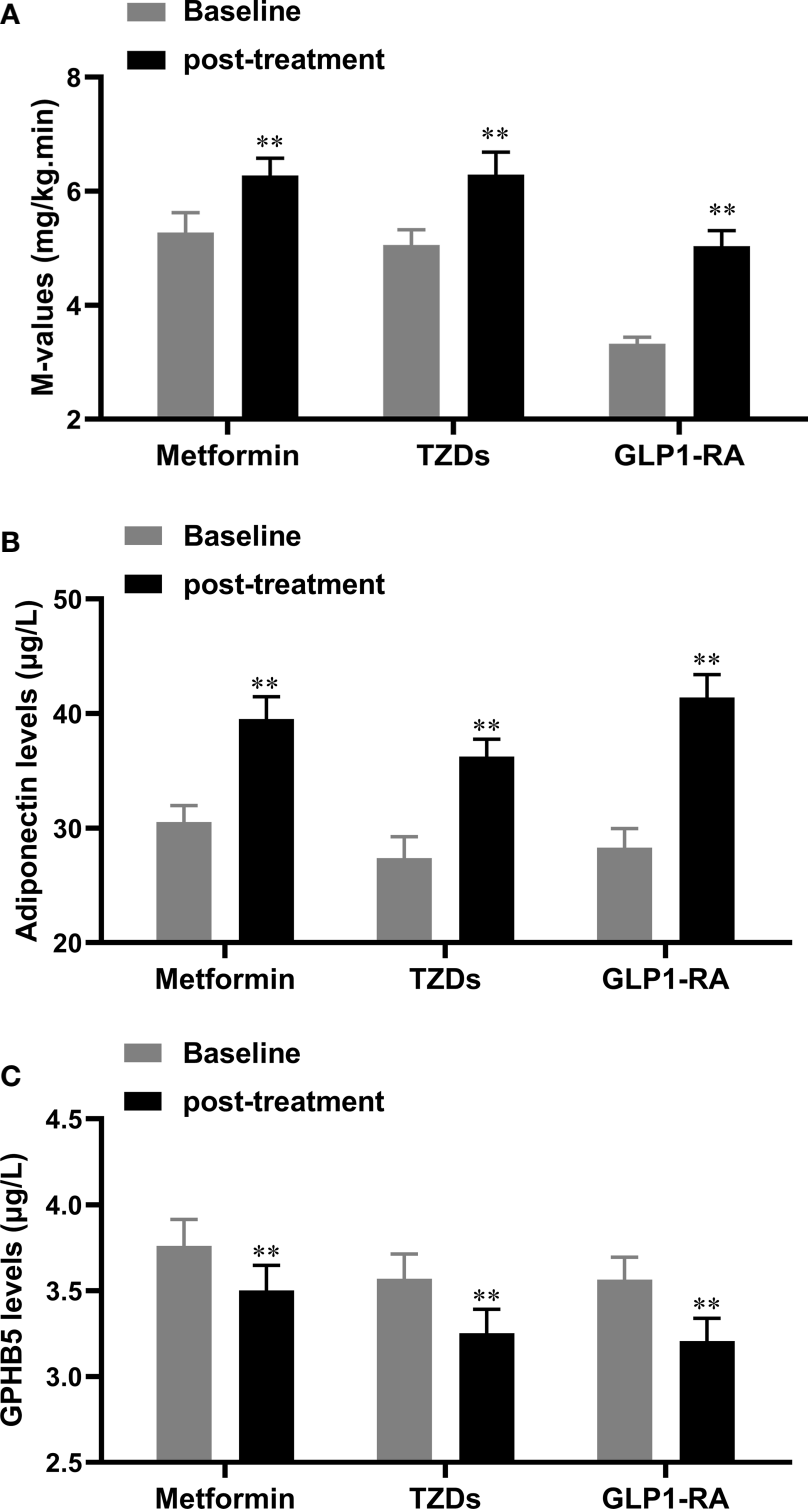
Serum GPHB5 and adiponectin levels in PCOS patients before and after treatment. **(A)** the changes of M-values during the EHC before and after treatment. **(B)** Circulating adiponectin levels before and after treatment. **(C)** Circulating GPHB5 levels before and after treatment. Data are means ± SD. * *p*<0.05, ** *p<* 0.01 vs. Baseline.

To observe the effect of anti-diabetic drugs on circulating GPHB5, three groups of PCOS women were treated with metformin, GLP-1RA (Lira), or TZDs for 24 weeks. As shown in **Table 5**, after 6 months of treatment, the WHR, FBG, 2-h BG, FIns, 2-h Ins, HOMA-IR, TC, LDL-C, FFA and AUCi in patients with PCOS were significantly lower than those at baseline in three groups. In addition, BMI, Fat%, TG and VAI were also significantly reduced in women treated with GLP-1-RA and metformin (**Table 5**). After treatment, AUCg was significantly lower in the GLP-RA and TZD groups, and BP was also significantly lower in GLP-1-RA group (**Table 5**). Furthermore, the EHC experiments found that all three treatments resulted in increased M-values (from 3.51 ± 0.72 to 5.07 ± 2.06 mg/kg/min for GLP-RA, from 5.27 ± 2.10 to 6.27 ± 1.84 mg/kg/min for metformin, and from 5.06 ± 1.58 to 6.29 ± 2.33 mg/kg/min for TZDs) (**Figure 8A**). In addition, after treatment with the three drugs, circulating Adipoq levels were significantly increased from 28.26 ± 9.64 to 39.54 ± 11.91 μg/L for GLP-RA, from 30.54 ± 8.59 to 39.52 ± 11.73 μg/L for metformin, from 27.39 ± 10.86 to 36.25 ± 8.83 μg/L for TZD μg/L (**Figure 8B**). These data suggest that these PCOS women had increased insulin sensitivity *in vivo* after treatment. Importantly, with the significant increase in circulating Adipoq levels and insulin sensitivity, the levels of circulating GPHB5 were decreased (from 3.79 ± 0.87 to 3.01 ± 0.89 μg/L for GLP-RA, from 4.02 ± 0.94 to 3.43 ± 1.01 μg/L for metformin and from 3.75 ± 0.82 to 3.11 ± 0.74 μg/L for TZD) (**Figure 8C**) after treatment with the three drugs, which further shows that GPHB5 is related to IR.

**Table 5:** Main clinical and metabolic features pre-and post-treatment with the anti-diabetes drug in PCOS women.

	GLP-1-RA		Metformin		TZDs	
Variable	Baseline	Post-treatment	Baseline	Post-treatment	Baseline	Post-treatment
BMI (kg/m2)	28.6 ± 4.2	26.3 ± 4.3**	25.0 ± 3.1	23.8 ± 2.9**	24.0 ± 3.1	23.1 ± 3.2
FAT (%)	41.0 ± 7.6	37.0 ± 5.8**	35.6 ± 6.3	34.0 ± 5.9*	34.1 ± 6.7	33.3 ± 8.4
WHR	0.90 ± 0.53	0.86 ± 0.52**	0.86 ± 0.07	0.83 ± 0.06**	0.85 ± 0.06	0.83 ± 0.06**
SBP (mmHg)	117.4 ± 11.2	111.9 ± 12.4*	112.2 ± 11.5	115.0 ± 8.1	115.6 ± 10.8	111.7 ± 11.5
DBP (mmHg)	74.6 ± 9.2	72.6 ± 8.4*	72.6 ± 6.7	73.3 ± 8.6	72.2 ± 11.6	72.0 ± 9.1
FBG (mmol/L)	5.47± 0.51	5.03 ± 0.51**	5.15 ± 0.58	4.97 ± 0.45**	5.23 ± 0.43	5.01 ± 0.35**
2 h-BG (mmol/L)	9.06 ± 3.20	7.06 ± 2.06*	7.81 ± 1.71	6.75 ± 1.67**	7.98 ± 1.99	6.98 ± 1.65**
FIns (mU/L)	30.9 ± 16.4	18.2 ± 12.1*	20.0 ± 13.2	14.0 ± 7.6**	15.2 ± 7.8	12.1 ± 7.2**
2 h-Ins (mU/L)	176.5(129.0 -337.2)	108.4 (73.1-173.4)**	122.3(84.0 -211.0)	88.4(58.2-133.8)*	129.5(91.8-179.2)	86.5(65.8-135.1)**
TG (mmol/L)	1.94 (1.50-2.50)	1.15 (0.81-1.79)**	1.51 (1.06-2.16)	1.03 (0.83-1.53)*	1.50 (1.23-1.82)	1.18 (1.01-1.69)
TC (mmol/L)	4.74 ± 0.81	4.14 ± 0.85**	4.53 ± 0.91	4.17 ± 0.78**	4.65 ± 1.03	4.18 ± 0.91**
HDL-C (mmol/L)	1.09 (0.98-1.26)	1.12 (0.99-1.29)*	1.20 (1.05-1.35)	1.25 (1.08-1.49)	1.17 (1.04-1.37)	1.33 (1.18-1.61)*
LDL-C (mmol/L)	2.78 ± 0.65	2.35 ± 0.63*	2.61 ± 0.86	2.25 ± 0.76**	2.64 ± 0.91	2.25 ± 0.75**
FFAs (μmol/L)	0.47 ± 0.19	0.38 ± 0.15**	0.64 ± 0.57	0.46 ± 0.17**	0.58 ± 0.31	0.43 ± 0.21**
AUCi	248.1(152.5-400.6)	173.6 (123.1-290.0)*	182.3(114.8-229.4)	149.9(96.4-181.8)*	190.6(141.7-261.0)	115.1(78.7-161.2)**
AUCg	13.40 ± 2.30	11.70 ± 2.30**	12.37 ± 2.37	11.95 ± 1.98	12.90 ± 2.20	11.40 ± 1.90**
HOMA-IR	6.61 (4.28-8.49)	3.30 (1.51-4.89)**	4.11 (2.59-5.04)	2.86 (1.82-3.84)**	3.02 (2.23-4.60)	2.46 (1.53-3.25)**
VAI	3.34 (1.86-4.81)	1.70 (1.13-3.31)**	2.31 (1.58-3.09)	1.47 (1.15-2.18)*	2.13 (1.70-2.96)	1.47 (1.11-2.53)
GPHB5 (µg/L)	3.56± 0.94	3.21 ± 0.95**	4.02 ± 0.96	3.40 ± 1.01**	3.75 ± 0.83	3.11 ± 0.75**
Adiponectin (µg/L)	28.3 ± 12.0	41.4 ± 14.4**	30.2 ± 8.6	39.6 ± 12.0**	27.4 ± 11.0	36.3 ± 9.0**

Values were given as means ± SEM or median (interquartile range). *p < 0.05, **p < 0.01 compared with baseline.

### Effect of antidiabetic drug treatment on sex hormone imaging in womenwith PCOS

The results of drug intervention showed that TEST and FAI levels decreased significantly after GLP-1RA intervention, while PRL increased significantly. Metformin treatment increased SHBG levels, but significantly reduced DHEA-S and FAI levels. TZD treatment increased SHBG, PRL and E2 levels, but significantly reduced TEST and FAI levels (**Table 6**). These results suggest that the three anti-diabetic drugs can affect the levels of sex hormones.

**Table 6:** Main hormone levels pre- and post-treatment with the anti-diabetes drug in PCOS women.

	GLP-1RA		Metformin		TZD	
Variable	Baseline	Post-treatment	Baseline	Post-treatment	Baseline	Post-treatment
SHBG (nmol/L)	30.1 (21.8 - 43.2)	37.3 (20.5 - 55.3)	31.8 (20.5 - 44.3)	46.7 (30.3 - 68.1)*	34.3 (25.4 - 50)	50.4 (39.7 - 68.6)*
DHEA-S (ug/DL)	276.9 ± 144.6	280.7 ± 163.2	251.4 ± 93.8	202.7 ± 77.5**	262.2 ± 140.9	254.8 ± 122.0
E2 (pg/ml)	41.1 (31.8 - 50.7)	42.2 (30.2 - 85.8)	185.7 (117.4 - 243.3)	190.5 (118.9 - 245.5)	135.8 (109.0 - 186.1)	186.1 (137.9 - 405.5)*
TEST (nmol/L)	1.92 ± 0.78	1.39 ± 0.53**	2.26 ± 0.83	2.02 ± 0.78	2.11 ± 0.96	1.51 ± 0.55**
LH (IU/L)	7.06 (4.80 -10.13)	3.73 (2.18 - 7.87)	6.24 (3.60 - 11.52)	4.70 (3.11 - 10.50)	8.03 (3.89 - 11.79)	5.48 (3.29 - 7.61)
FSH (IU/L)	5.83 (4.73 - 6.74)	5.88 (4.10 - 7.53)	7.18 (5.71 - 8.98)	7.39 (5.81 - 8.59)	6.02 (5.61 - 6.93)	5.90 (4.96 - 6.84)
PRL (μIU/L)	302.1 ± 165.4	377.6 ± 225.9*	355.6 ± 210.3	369.9 ± 205.7	300.8 ± 127.5	406.5 ± 226.2**
FAI	6.27 (3.60 - 9.12)	3.31 (2.03 - 6.47)**	6.61 (3.97 - 11.22)	3.87 (2.50 - 6.62)*	5.57 (3.82 - 8.72)	3.05 (1.99 - 4.43)**

Values were given as means ± SEM or median (interquartile range). *p < 0.05, **p < 0.01 compared with baseline.

## Discussion

In the current study, we find that GPHB5 is related to metabolic disorders, IR and PCOS through internet data analysis. We also find that the expression of GPHB5 mRNA is significantly increased in diet-induced obese and diabetic mice and rats with PCOS. In a population cohort study, we examined the circulating GPHB5 levels in women with IR and PCOS and compared them with those in normal individuals. The results showed that in women with IR and PCOS, the levels of circulating GPHB5 increased significantly compared with normal individuals, and corresponded to the reduced M-values and Adipoq levels, an insulin sensitive factor. In addition, correlation analysis showed that the increased circulating GPHB5 level was positively correlated with BMI and HOMA-IR but negatively correlated with the M-value of EHC and adiponectin, suggesting that the increase in GPHB5 levels may promote metabolic disorder and IR. In addition, GPHB5 was also found to be significantly correlated with FAI. These results suggest that GPHB5 may be a biomarker of IR and PCOS. However, a mouse study showed that GPHB5 overexpression improved diet-induced obesity, reduced the levels of blood glucose, triglycerides, cholesterol and insulin, and increased the levels of T3 and T4 (17). In our study, the strong association of circulating GPHB5 with IR and metabolic disorders is confirmed. However, unlike previous studies, our results show that the increase in GPHB5 may promote IR and metabolic disorders. Moreover, elevated circulating GPHB5 levels did not lead to increased T3 and T4 levels. Unlike the Macdonald et al. study, the reason for the inconsistent results is not unknown. This may be due to the differences between humans and mice or the differences in experimental conditions and methods. These results need to be further replicated.

Based on the above findings, the further question is whether GPHB5 is regulated by blood glucose or insulin levels. Therefore, we first performed the OGTTs in the study populations. We found that hyperglycemia and hyperinsulinemia caused by glucose challenge did not lead to changes in circulating GPHB5 levels. To exclude the interference of hyperglycemia, we performed an EHC study, the gold standard for evaluating insulin sensitivity (26), and measured circulating GPHB5 levels in women with IR and PCOS and normal individuals. In this study, blood glucose was clamped at the basal level and a hyperinsulinemia was produced by exogenous insulin infusion. However, short-term hyperinsulinemia did not lead to changes in circulating GPHB5 levels. These results suggest that the secretion and release of GPHB5 may not be regulated by changes in blood glucose and insulin levels in the short term.

Metformin, GLP-1RA, and TZDs are widely used antidiabetic drugs that increase insulin sensitivity and improve metabolic disorders (20). Metformin has been reported more in the treatment of PCOS, while GLP-1RA and TZDs are reported less in the treatment of PCOS women (2, 29, 30).

In the current study, we treated PCOS women with three drugs for six months for the first time and observed the levels of blood biochemistry, sex hormones, circulating Adipoq and GPHB5. As expected, GLP-1RA and metformin significantly reduced body weight in women with PCOS, while rosiglitazone had no effect on body weight. These results suggest that GLP-1RA and metformin have the advantage of BW loss in obese women with PCOS. Importantly, although TZD treatment did not reduce BMI, WHR was significantly lower than before treatment. Therefore, it has been suggested that TZDs treatment leads to fat redistribution and reduces abdominal fat (31). In addition, BP was significantly lower in women treated with GLP-1RA than before treatment, suggesting that it has a certain anti-hypertensive effect. This result is similar to previous reports in T2DM patients (32). However, its mechanism remains unknown and needs further study.

The results of the EHC experiment showed that M-value increased significantly after treatment with the three drugs. In addition, HOMA-IR also decreased significantly. Therefore, these data suggested that insulin sensitivity in the three groups was improved. After 6 months of treatment, with weight loss and the improvement of metabolic disorder and IR, the levels of circulating Adipoq increased significantly, while the level of GPHB5 decreased significantly. These findings further indicate that GPHB5 is related to IR and may be a biomarker opposite to Adipoq.

Although affected by many factors, IR and high androgen levels are considered to be causes of PCOS (33, 34). Therefore, we observed changes in sex hormone levels in women with PCOS after six months of treatment with the three drugs. Our results showed that TEST levels decreased significantly after GLP-1RA and TZDs treatment, while there was no significant change in the metformin treatment group. These findings suggest that GLP-1RA and TZD, but not metformin, can inhibit androgen production. An *in vitro* study found that TZD can inhibit the activities of two key enzymes of androgen biosynthesis, P450 and 3βHSDII, and thus it may inhibit androgen synthesis (35). In addition, a previous study in cells and animals has found that GLP-1 receptors and androgen interact at the transcriptional level (36). However, the mechanism by which GLP-1RA and TZD decrease androgen production remains unknown. Furthermore, the relationship between GPHB5 and androgen has not been reported. In the current study, we find that circulating GPHB5 levels were significantly correlated with FAI, DHEAS and SHBG. Therefore, further research is needed.

The main advantages of the current study are as follows: 1) It is based on two large study cohorts from a real screening environment, including women with PCOS and IR; 2) to exclude the interference of gender, we chose women as participants; 3) To investigate the relationship between GPHB5 and blood glucose and insulin, we conducted several intervention experiments, including the EHC; 4) Three drugs were used to treat patients with PCOS, and the changes in various indexes and GPHB5 levels were compared before and after treatment. However, our study also has some limitations: 1) although the overall size of the study population was large, the number of patients was still relatively limited after grouping, a feature commonly encountered in cohort studies; 2) Our research was only carried out in a Chinese population, so it should be applied it to other ethnic groups with care; 3) because of ethical limitations, there is no placebo control in the drug study; 4) drug treatment did not use random groupings, but self-control before and after treatment.

In conclusion, circulating GPHB5 levels were elevated in PCOS and IR populations and associated with glucose and lipid metabolism disorders, obesity and IR. Our data suggest that GPHB5 may be a promising risk stratification marker to help identify high-risk candidates for PCOS and IR. However, further population-based studies are needed to replicate and confirm these findings.

## Data availability statement

The original contributions presented in the study are included in the article/Supplementary Material. Further inquiries can be directed to the corresponding authors.

## Ethics statement

The studies involving human participants were reviewed and approved by the Chinese Clinical Trial Registry (CHICTR- OCS-13003185). The patients/participants provided their written informed consent to participate in this study. The animal study was reviewed and approved by the ethical committee of Chongqing Medical University. Written informed consent was obtained from the individual(s) for the publication of any potentially identifiable images or data included in this article.

## Author contributions

YW, TX, XX, HZ, SG, KL, SQ, YH, RL, and LZ collected specimens, analyzed, and interpreted the data. HL and LL coordinated the study and revised the manuscript. GY provided statistical analysis. ZL and JH are the guarantors of this work and as such, had full access to all of the data in the study and take responsibility for the integrity of the data and the accuracy of the data analysis. All authors contributed to the article and approved the submitted version.
